# The evolution and future of influenza pandemic preparedness

**DOI:** 10.1038/s12276-021-00603-0

**Published:** 2021-05-06

**Authors:** Walter N. Harrington, Christina M. Kackos, Richard J. Webby

**Affiliations:** 1 Department of Infectious Diseases, St. Jude Children’s Research Hospital, Memphis, TN USA; 2 St. Jude Children’s Research Hospital, Graduate School of Biomedical Sciences, Memphis, TN USA

**Keywords:** Influenza virus, Infectious diseases

## Abstract

The influenza virus is a global threat to human health causing unpredictable yet recurring pandemics, the last four emerging over the course of a hundred years. As our knowledge of influenza virus evolution, distribution, and transmission has increased, paths to pandemic preparedness have become apparent. In the 1950s, the World Health Organization (WHO) established a global influenza surveillance network that is now composed of institutions in 122 member states. This and other surveillance networks monitor circulating influenza strains in humans and animal reservoirs and are primed to detect influenza strains with pandemic potential. Both the United States Centers for Disease Control and Prevention and the WHO have also developed pandemic risk assessment tools that evaluate specific aspects of emerging influenza strains to develop a systematic process of determining research and funding priorities according to the risk of emergence and potential impact. Here, we review the history of influenza pandemic preparedness and the current state of preparedness, and we propose additional measures for improvement. We also comment on the intersection between the influenza pandemic preparedness network and the current SARS-CoV-2 crisis. We must continually evaluate and revise our risk assessment and pandemic preparedness plans and incorporate new information gathered from research and global crises.

## Influenza: Learning to plan for pandemics

The long history of combating and planning for influenza pandemics should inform the fight against novel coronaviruses such as SARS-Cov-2. Richard Webby and co-workers at St. Jude Children’s Research Hospital in Memphis, USA review the history of preparing for influenza pandemics, including the global influenza surveillance network set up by the World Health Organization (WHO) in the 1950s. The 2009 H1N1 pandemic prompted WHO and the US Centers for Disease Control and Prevention to develop more detailed risk assessment tools drawing on laboratory research, genomics, industrial vaccine development, and surveillance of emerging animal strains that might transfer to humans. These tools and experience are proving successful in containing the H7N9 influenza that emerged in 2013, and could serve as models for managing coronaviruses, whose pandemic potential has only become apparent in the past two decades.

## Introduction

**Fig. 1:**
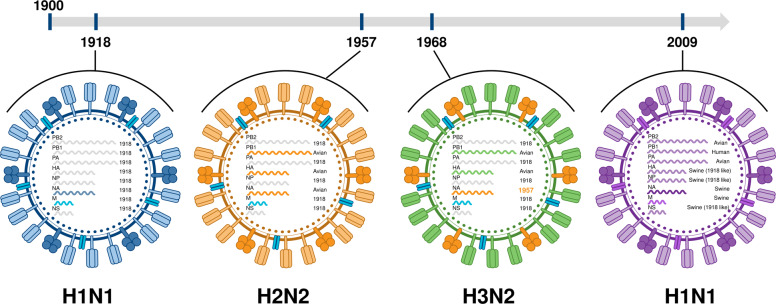
History of influenza pandemics. There have been four influenza pandemics since the turn of the 20th century, occurring in 1918 (H1N1), 1957 (H2N2), 1968 (H3N2), and 2009 (H1N1). This timeline shows the temporal and genetic reassortment relationships among each of the pandemic influenza subtypes.

Influenza has plagued humanity for centuries. Influenza A and B viruses are endemic in humans and responsible for annual epidemics across the globe. In addition to annual epidemics, influenza A viruses were responsible for four pandemics from 1918 to 2009 (Fig. 1). Aquatic avian species are the natural reservoir of influenza A viruses, which have adapted to infect many other animals, including swine, domestic poultry, dogs, horses, and others^1^. This adaptation has provided ample hosts from which zoonotic strains may transmit to humans. The influenza virus has eight gene segments, each of which can be substituted through genetic reassortment when two or more viruses infect the same cell to ultimately produce a novel variant^2^.

Host diversity and gene segmentation were responsible for the 2009 H1N1 pandemic, in which a series of reassortment events among avian, human H3N2, and swine H1N1 viruses via swine produced a novel influenza strain that was transmitted among humans (Fig. 1)^3^. Although the 2009 H1N1 pandemic was comparatively mild in most age groups, the events that led to its emergence demonstrate the unpredictability of influenza pandemics. Furthermore, the 2009 H1N1 pandemic highlighted the need for a comprehensive framework to evaluate influenza strains for their likelihood of emergence and public health risk. Accordingly, both the World Health Organization (WHO) and United States Centers for Disease Control and Prevention (CDC) created scoring systems to evaluate prepandemic influenza viruses in three primary categories: (1) properties of the virus, (2) attributes of the human population, and (3) viral ecology and epidemiology^4,5^.

We review our tangled history with influenza that has influenced our pandemic response and examine our current methods of preparedness, including influenza surveillance and current risk assessment criteria. Furthermore, we evaluate how the CDC and WHO pandemic risk assessment tools affected our response to H7N9 outbreaks after the 2009 H1N1 pandemic, how these tools may be used to manage the ongoing SARS-CoV-2 pandemic, and the future outlooks of and goals for influenza pandemic preparedness.

## Influenza pandemic and preparedness history

It is generally acknowledged that influenza pandemics have long been a part of human history. Reports describe bouts of influenza-like respiratory disease as far back as 412 BC, with fifteen likely influenza pandemics occurring after the 1700s when descriptions became more reliable^6^. It was not until the 1930s, however, that the causative agent, influenza virus, was isolated with Koch’s postulates being fulfilled^7^. The improvements in virus growth and handling that followed allowed for mass production and quantification, which were critical steps toward the subsequent development of a vaccine. This vaccine was followed in the 1940s with studies conducted by the United States Military. The overall vaccination strategy used in these seminal studies is soberingly similar to that used for most influenza vaccines today. Virus harvested from the allantoic fluid of embryonated hen eggs inoculated 48 h prior was concentrated, inactivated, and administered subcutaneously. The first studies of this vaccine were successful, and the immunization of military personnel^8^ represented the first rationally designed pharmaceutical intervention against influenza. While sporadically used in intervening years, further studies of the same vaccine in 1947 were unable to identify any positive benefit, with the authors postulating the failure due to “the lack of sufficient antigenic crossing between strains of virus in the vaccine and the prevalent strain responsible for the epidemic”^9^. In response to this growing realization of the variable nature of influenza, in 1947, the Global Influenza Programme (GIP) was established within the WHO^10^. This initiative started with the designation of the World Influenza Centre at the Medical Research Council of Great Britain. The impetus for establishing this program was articulated as being driven by memories of the 1918 pandemic, the highly infectious nature of the disease, and the speed of its spread and economic impact on countries. Correspondingly, the stated objectives of the GIP were to plan against possible recurrence of a pandemic, devise control methods, and limit economic impact from which influenza pandemic preparedness was essentially born. A further review of the driving forces behind GIP implementation shows how astute the supporters of these early efforts were and how their statements still hold true. In his announcement of the GIP, Payne from the WHO Division of Communicable Disease Services, although it was unclear at that time how influenza maintained itself between seasonal epidemics, surmised that (1) successful vaccination against influenza depends on knowledge of the virus causing epidemics, (2) continuous vigilance is necessary to detect new and potentially dangerous strains of virus at the earliest possible moment, and (3) epidemiological reports can be correctly interpreted only in terms of laboratory studies of the viruses responsible^10^. Despite the WHO influenza monitoring system (now called the Global Influenza Surveillance and Response Network (GISRS)) currently having been expanded to 144 laboratories in 122 countries^11^, every one of these tenants rings true today.

## Influenza preparedness

### Surveillance

**Fig. 2:**
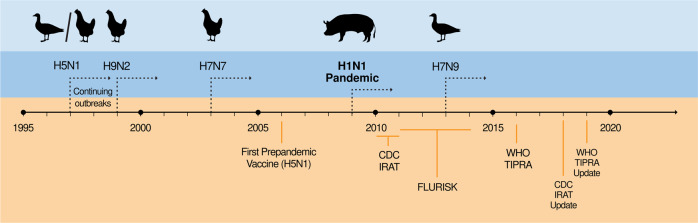
Prepandemic influenza and preparedness. A combined timeline indicating the temporal relationships between emerging potential pandemic strains (with accompanying animal reservoirs) and steps taken to improve pandemic preparedness. Dashed arrows indicate continuing outbreaks for each strain after they first emerged.

A key component of pandemic preparedness is the ability to detect novel influenza strains as they emerge in the human population. These strains emerge through spillover events from animal reservoirs to humans and must be detected as quickly as possible before sustained human-to-human transmission^12^. Within the last 20 years, multiple influenza zoonotic events have been detected (Fig. 2); therefore, the surveillance of the influenza strains circulating in humans and animals, particularly in avian and swine species, is crucial for early detection, an endeavor supported by the GISRS and other international agencies such as the Food and Agricultural Organization of the United Nations.

As innovations such as hunting, animal domestication, industrialization, and sprawling urbanization have increased human interactions with various animal species and their accompanying zoonoses, the likelihood of spillover events has consequently also increased. For influenza, the presence of large, live-bird markets provides an environment for such interactions with influenza viruses. In 1997, a fatal respiratory disease developed in a child in Hong Kong and was caused by an H5N1 avian influenza virus^13^. Seventeen additional H5N1 infections were detected later that year, killing one-third of those infected^14^. Culling poultry, cleaning, and monitoring the live-bird markets in Hong Kong eliminated new human cases of H5N1 until its reemergence in 2003^14,15^.

Culling is currently the most effective strategy to reduce or prevent the transmission of influenza viruses in poultry, although large-scale vaccination efforts may also curb their spread^16^. The surveillance of live-bird markets and other high-risk areas with similar conditions is critical for understanding the geographic distribution of circulating influenza viruses, a key component of influenza pandemic risk assessment. International, national, and academic stakeholders must also continue to synchronously guide global influenza research and public health responses, sharing reagents and other resources to support surveillance and research on influenza virus evolution and transmission.

### Laboratory examination

Basic research on influenza pathogenesis, evolution, and host interactions lays the foundation for all pandemic preparedness strategies. Key insights into transmission and natural reservoirs directly affect the surveillance and risk assessment of emerging strains^4,17,18^. Two research fields have expanded our understanding of influenza infections: viral pathogenesis and host immune responses^19,20^.

Laboratory research can reveal crucial insights into the pathogenesis of different influenza strains and guide assessments of risk. The reassortment of genetic material from influenza strain coinfections is a driver of pandemic virus emergence^21–23^. Although hemagglutinin (HA) and neuraminidase (NA) glycoproteins on the surface of the virus are generally considered drivers of influenza infectivity and pathogenesis, studies have shown that internal genes (e.g., polymerase genes^24,25^) can confer disease severity characteristics to novel influenza strains^26,27^.

Pathogenesis studies are facilitated through in vitro and in vivo laboratory models that provide key information on viral traits and host immune response. For example, avian and mammalian culture methods are used as surrogate indicators for human infection and vaccine protection^28,29^. In addition, the ferret influenza model is leveraged to predict pathogenicity and transmissibility in humans, even discerning between direct and aerosol-contact modes of transmission^30,31^.

### Risk assessment tools

With much focus at the time on avian H5N1 viruses, the 2009 H1N1 pandemic illuminated the need for a standardized system to assess the potential risks of emerging and circulating influenza strains and to allocate the limited time and financial resources available to research, prevention, and treatment. After the 2009 pandemic, two major risk assessment tools were developed to meet this need, combining insights from surveillance and laboratory work to develop a general framework for overall potential pandemic risk. These tools are careful to state that they do not predict which pandemic strain will emerge next but rather outline systematic methods for deciding research and funding priorities for emerging strains. Both require input from subject matter experts who score individual elements for a given virus.

In 2010, the CDC created the Influenza Risk Assessment Tool (IRAT) to guide strategic decisions for future influenza pandemic preparedness^32^. The IRAT evaluates prepandemic influenza viruses not currently circulating in humans based on ten characteristics, weighted according to perceived pandemic risk: (1) genomic variation, (2) receptor binding, (3) transmission in animal models, (4) antiviral susceptibility, (5) immunity in humans, (6) disease severity, (7) antigenic similarity to currently circulating strains in humans, (8) global distribution in animals, (9) animal species infected, and (10) human infections^32^.

In 2016, the WHO developed the Tool for Influenza Pandemic Risk Assessment (TIPRA) to estimate the pandemic risk of emerging influenza strains^4^. The TIPRA was modeled closely after the IRAT, with 9 risk elements: (1) receptor binding properties, (2) genomic characteristics, (3) transmission in animal models, (4) susceptibility to antiviral treatment, (5) human infection, (6) disease severity, (7) population immunity, (8) geographic distribution in animals, and (9) infections in animals. The TIPRA estimates the risk of sustained human-to-human transmission of emerging viruses.

**Fig. 3:**
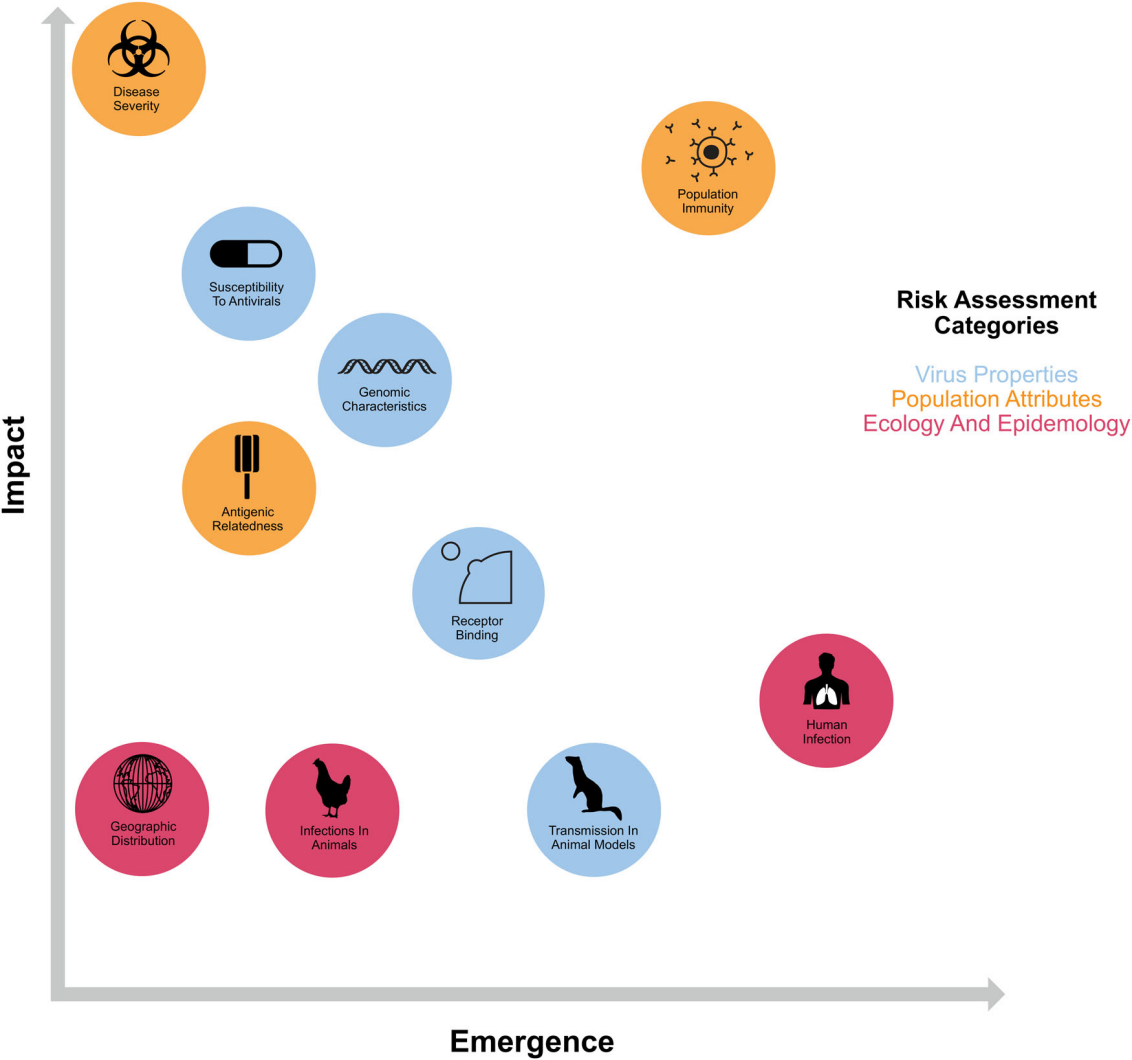
Weighted risk assessment elements to estimate emergence and impact risks for prepandemic influenza strains. Schematic graph indicating the relative weights that each of the risk elements defined by the IRAT and TIPRA contribute to the overall assessment scores for emergence and impact risk of an emergent influenza strain.

Both the IRAT and the TIPRA are multielement additive models with weighted categories, with each weight determined through expert consultation and discussion. Figure 3 is a graphical representation of the relative relationships between elements from the IRAT and TIPRA. Additional risk assessment tools, such as FLURISK, have also been proposed^33^. Below, we discuss the strengths and weaknesses of these elements and offer suggestions for improvement.

### Defining risk assessment characteristics

The risk elements defined by the IRAT and TIPRA are grouped into three general categories (Fig. 3): (1) viral properties, (2) human population attributes, and (3) viral ecology/epidemiology in animal hosts. Furthermore, they encompass two dimensions of prepandemic strains: (1) their likelihood of developing into a pandemic strain (emergence) and (2) their level of severity in a pandemic (impact).

### (1) Viral properties

#### Genomic characteristics

When assessing the pandemic risk of an influenza virus, genomic characteristics can help predict how viral strains will behave before the behavior is observed in the field. General patterns of virulence and transmission are associated with specific genetic markers in the influenza genome^26^. For example, if a virus is markedly divergent from endemic strains, especially in the HA or NA genes, it has a higher probability of presenting novel antigens to the population, making pandemic spread more likely. If novel viruses are already circulating in mammals, they may contain genetic signatures associated with human infectivity and/or virulence and would score more highly in this element. Such considerations should be factored into assessing the potential pandemic risk of novel influenza viruses.

### Receptor binding

Receptor binding is a key barrier that avian, and possible mammalian, influenza viruses must overcome to infect and transmit in humans^34^. Influenza viruses use cell surface glycans with sialic acid to bind and enter cells. Most of these cell surface glycans on human airway epithelial cells contain α2,6 linkages, whereas those on avian cells typically contain α2,3 linkages^35^. Therefore, influenza strains well adapted to α2,3 linkages must gain mutations that facilitate binding to α2,6 linkages to cross the species barrier into humans. Swine species provide a prime environment for the selection of receptor binding mutations, as cells in the trachea of swine contain a mixture of α2,3 and α2,6 linkages on their surface^36,37^. Receptor binding can be assessed by glycan-binding assays, but recent data suggest that primary human cell models are more physiologically relevant indicators of receptor binding properties^38^. These properties are factored into pandemic risk potential estimates.

#### Transmission in animal models

The ferret model is particularly important for the prediction of human transmissibility, measuring both direct- and aerosol-contact–mediated transmission^39,40^. In the direct-contact model, infected and noninfected ferrets are housed together, sharing space, food, and water. In the aerosol-contact model, infected and noninfected ferrets are housed near each other to permit airborne transmission of respiratory droplets without direct contact. These two models can provide crucial information about whether prepandemic strains are likely to demonstrate sustained human-to-human transmission. For example, a strain that transmits via direct and aerosol contact in an animal model has a higher risk potential of sustained transmission than one that transmits via direct contact alone.

#### Susceptibility to antiviral treatment

Once a novel influenza virus reaches the pandemic level, the determination of appropriate treatment options is vital^41,42^. However, novel therapeutics against influenza strains resistant to current treatment options typically take years of research and clinical trials to produce. Thus, susceptibility to antiviral treatment should be a major factor for risk assessments of prepandemic strains. This criterion is important to guide the allocation of research and resources towards prepandemic strains that have the highest chance of presenting major treatment challenges should they cross the host-species barrier.

### (2) Human population attributes

#### Population immunity

Influenza pandemic risk assessments must also consider population immunity to emerging strains. Antibody responses to HA and NA surface glycoproteins are primary markers of protective immunity^43^. Therefore, emerging influenza viruses that are antigenically similar to seasonal influenza strains endemic in humans are more likely to be hindered by population immunity. Conversely, influenza strains originating from animal reservoirs with novel HA or NA subtypes are more likely to cause pandemics.

#### Human infection

Unfortunately, our best predictor of human infection potential is provided by evidence of past human infection. As such, viruses such as the A/goose/Guangdong/1/96-lineage H5N1 viruses, which have caused hundreds of human infections with a mortality rate of ~60%^44^, are postulated to be of higher risk than an H14 virus that has never been found in humans. However, despite circulating for over 20 years, human-to-human transmission of H5N1 is rare, and infection is typically acquired through close contact with infected birds, lowering its pandemic risk^45–47^. Thus, quantities of past human infections do not necessarily correlate directly with the ability for pandemic spread.

#### Antigenic relatedness

A large determinant of our ability to control the spread of pandemic influenza strains is the availability of vaccines to increase population immunity and limit the number of naïve hosts for infection. However, changes to influenza vaccines typically require at least 6 months from conception to final licensing and distribution^48^. Although SARS-CoV-2 vaccines are being produced in record time, with candidates moving from preclinical testing to phase III trials in under 1 year^49^, countless individuals may become infected and succumb to the virus even with expedited vaccine production. Existing seasonal or pandemic influenza vaccine stockpiles may minimize the impact of an emerging strain if they are sufficiently antigenically related to the emerging virus. As the antigenic distance between seasonal and pandemic strains increases, the efficacy of existing vaccines decreases^50^. This particular element is one of the few present in only one of the risk assessment tools (the IRAT).

### (3) Virus ecology/epidemiology in animal hosts

#### Infection in animals

Many different animal models are used in influenza research, including mice, chickens, pigs, hamsters, guinea pigs, and ferrets^51^. The ferret is the gold standard to predict and/or study how influenza strains behave in humans because ferrets recapitulate human influenza infections and have a prominence of α2,6 linkages on their airway epithelial cells^31,52,53^. The infectability of ferrets provides some indication of how well a virus is adapted to infect humans.

As interaction between an infected animal population and humans is a clear requirement for pandemic virus emergence, the host animal reservoir of a virus is an important consideration. For example, a virus circulating widely in backyard poultry is more likely to come into contact with a human host than a virus in a population of wild birds.

#### Geographic distribution in animals

Geographic distribution in animals considers both the location and the speed of spread of prepandemic strains. Assessing viral locality indicates how easily a virus can be contained. Strains that are only locally distributed exhibit slow to moderate spread that occurs for understandable and predictable reasons (e.g., animals are moved from one farm to another) or are contained in single animal populations have a low to moderate risk of causing pandemics. Strains that are distributed widely, show unpredictable spread to other populations, or spread rapidly have a high risk of causing pandemics. A virus with a wider geographic distribution is also more likely to encounter a susceptible human host.

#### H7N9: a test case

H1, H2, and H3 are the only influenza subtypes known to cause pandemics. However, recent outbreaks of the H5, H7 and H9 subtypes have raised alarms regarding their pandemic potential^54–56^. Therefore, we use the recent H7N9 outbreaks to evaluate our current pandemic risk assessment and assess the strengths and weaknesses of our current level of preparedness.

H7N9 was first isolated in humans in March 2013 and has since caused 1,567 human cases and 615 deaths, a concerning mortality rate^57^. H7N9 is a novel reassortment avian lineage virus that most likely acquired internal genes from an H9N2 avian virus, an HA gene from a duck H7N3 virus, and an NA gene from an H7N9 virus circulating in migratory birds^26^. Human H7N9 outbreaks occurred in six waves in China, the latest occurring in 2017, with most cases linked to direct exposure to birds^58^.

Early identification and risk assessments led to a quick response from the research community. The novel strain responsible for the first case in March 2013 was reviewed according to the pandemic risk assessment model by August of that year^26^, and candidate prepandemic H7N9 vaccines were identified and recommended by the WHO by the summer of 2013^59^. Subsequently, many researchers began studying H7N9 in vitro and in vivo.

The H7N9 genome contains mutations that increase human receptor binding and replication, a lack of multiple basic amino acids at the HA cleavage site (leading to low pathogenicity and undetected spread in birds), and an internal NS-associated gene constellation from H9N2 that led to high pathogenicity when reassorted into the highly pathogenic H5N1 outbreak in 1997^26^. These mutations may contribute to its high pathogenicity and mortality rate in humans.

H7N9 emergence was a driving factor for developing the TIPRA, and by early 2018, this tool was used to evaluate the risk of this and similar prepandemic strains^4,32^. H7N9 scored highest for risk potential among 14 animal-origin viruses, ranking high for both emergence and impact risk^32^. H7N9 is concerning for its pandemic risk, and many resources in research and surveillance are consequently dedicated to this strain.

Since 2013, the H7N9 virus has diverged into two major, antigenically distinct genetic lineages. Fortunately, there is evidence to suggest that stockpiled prepandemic vaccines developed against the early 2013 H7N9 viruses will be effective against each lineage. The vaccination of humans with these stockpiled vaccines elicited antibodies that were able to mediate antibody-dependent cellular cytotoxicity and neuraminidase inhibition of the antigenically drifted A(H7N9) viruses^60^. H7N9 is not yet capable of sustained human-to-human transmission, a key determinate in causing pandemics. Since its first infection in humans, many resources have been dedicated to this response to this virus, resulting in a vast knowledge base. With prepandemic vaccines stockpiled, cell line and animal models optimized, and the surveillance system on alert, we are relatively confident that a quick response can be mounted should H7N9 gain the ability of sustained human-to-human transmission. However, we should always be evaluating and updating our current pandemic plans as we gain new information.

## Future

### The future of influenza pandemic preparedness: goals and outlooks for the next decade

**Fig. 4:**
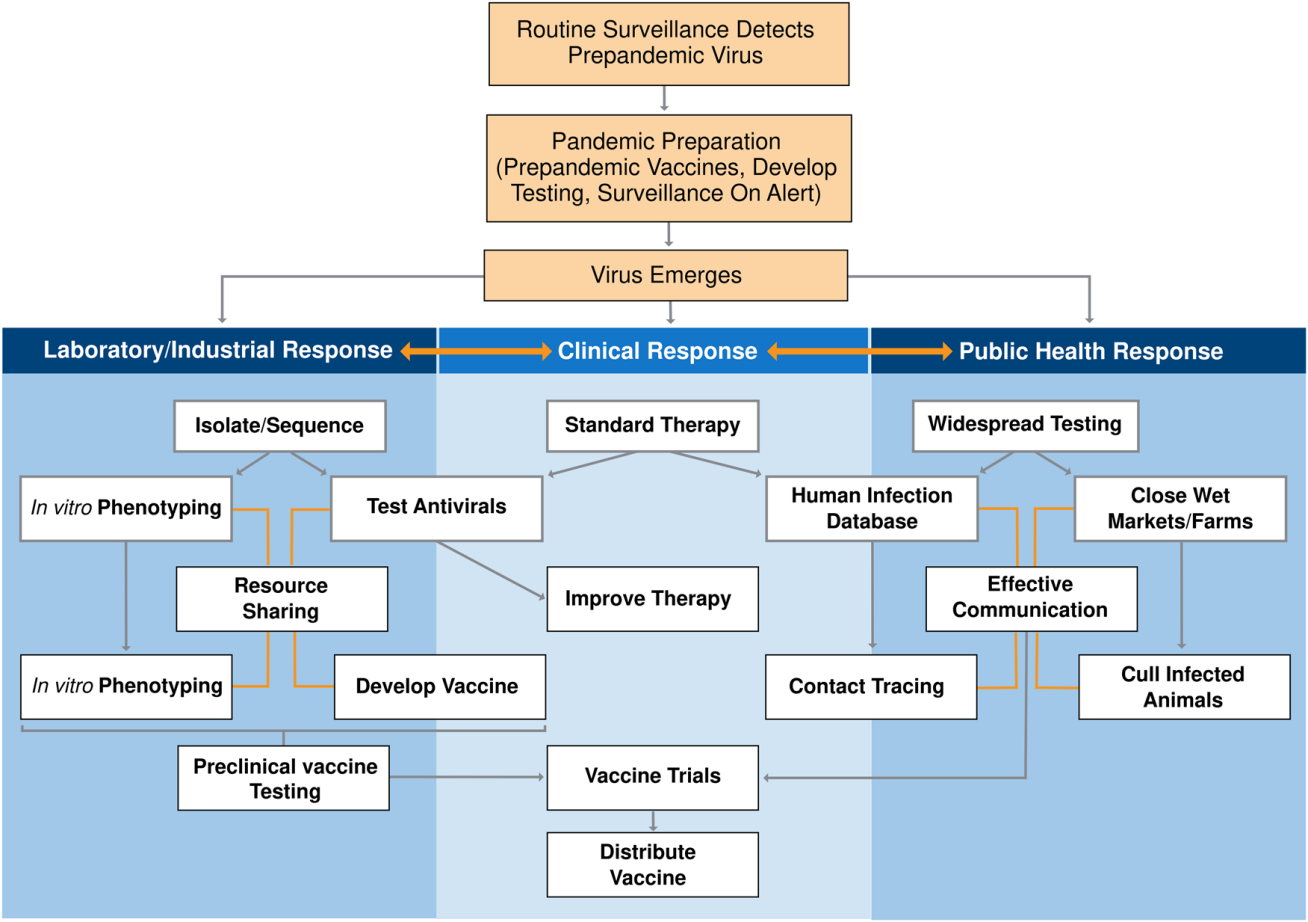
Ideal pandemic response workflow. A schematic workflow of the ideal pandemic response from prepandemic surveillance detection to widespread vaccine distribution.

As we move into the future of influenza pandemic preparedness, we must continue to improve metrics for pandemic risk assessments and our ability to respond to and mitigate such events. Areas for continued investment include surveillance and coordination among research laboratories, risk assessment criteria, vaccines and therapeutics, and clear and cohesive public health strategies and messaging. Fig. 4 presents an integrated system workflow that incorporates current and proposed elements of influenza pandemic preparedness and response.

### Surveillance

Effective influenza pandemic responses rely on rapid detection and identification of emerging strains. Expanded sampling of animal species and human infections increases the likelihood that such strains will be detected early. Continued collaboration among human and animal health influenza surveillance centers will ensure that reagents are appropriately shared, developed, and validated. Furthermore, these centers must be poised to respond when prepandemic influenza strains are identified, including maintaining adequate containment facilities and identification reagents.

To ensure the ability of these centers to identify and respond to emerging strains, surveillance efforts should be broadened. Increased funding of pandemic preparedness efforts would allow for expanded sampling of zoonotic viruses, as well as the development of reagents and techniques to more precisely assess the risk posed by these viruses. Furthermore, increased support of such efforts would result in a larger arsenal of countermeasures, such as therapeutics, which may be deployed when high-risk prepandemic influenza strains are identified. Funding for surveillance is, shortsightedly, very difficult to maintain long term in the absence of emerging events.

### Laboratory research

The study of differential genetic mutation rates among influenza RNA-dependent RNA polymerases may be particularly relevant to pandemic risk assessments. Although the overall mutation rate of influenza has been explored, new methods can compare mutation rates among different influenza strains^61^. Higher mutation rates are linked to greater population diversity and thus have higher potential to evade host immune responses and develop resistance to antivirals^62,63^. Tools that can quickly identify strains with high mutation rates or RNA-dependent RNA polymerases with a high propensity for introducing mutations during replication can inform the evaluation of strains for their pandemic potential. Our ability to predict pandemic risk also requires a far larger arsenal of molecular markers of viral phenotypes. The sequencing of viruses is becoming increasingly cheaper and of higher throughput, and the more information that we can glean from sequencing data alone, the stronger our preparedness will be.

### Assessment criteria

As our understanding of influenza viral dynamics and disease pathology grows, we must use this knowledge to expand and refine the assessment criteria for evaluating prepandemic strains. Although the risk assessment categories comprehensively represent our current understanding of the factors contributing to emerging influenza strains, other viral and host factors may play an underappreciated role in influenza pandemic probability.

For example, we suggest that the acid stability variability between HAs of different subtypes should be considered as a new factor in the assessment criteria. During the viral life cycle, HA binds to the terminal sialic acids of host cell receptors for virion internalization to initiate infections. HA is translated, glycosylated, and acetylated into the trimeric precursor protein HA0, which is functionally inactive and cannot facilitate endocytosis or macropinocytosis for viral entry into cells^64,65^. The HA0 precursor is activated via cleavage into the HA1/HA2 complex, a high-energy complex that undergoes conformational changes at low pH to enable membrane fusion^66^. This pH-dependent conformational change suggests that the stability of HA subtypes varies in differing environments. Accordingly, the pH at which HA0 cleavage occurs differs among subtypes. Influenza subtypes endemic in humans are relatively stable, whereas emerging subtypes in humans have a higher pH of activation. Swine isolates have activation pH values that span those found in human isolates, and avian isolates have wide-ranging activation pH values. Mutations that alter HA stability occur across several subtypes in different species^67^. Therefore, pH-dependent HA stability may have important implications for influenza virion transmission within mammalian hosts. In support of this, multiple studies in mice and ferrets revealed that the stabilization of H5 contributes to gain-of-function airborne transmission of H5N1 in ferrets and facilitates enhanced upper respiratory tract replication^68–70^.

Although the complete mechanism of pH-dependent HA stabilization is unknown, environmental stability most likely plays a role. A lower activation pH of H5N1 is associated with greater environmental persistence and may prevent premature extracellular activation in the mammalian respiratory tract. To fully understand this mechanism and the association of HA stability with host range, further studies are needed. However, early findings on HA stability in viral adaptation to human hosts suggest that HA activation pH should be included in the influenza risk assessment framework.

### Vaccines and therapeutics

Our ability to combat future influenza pandemics lies largely in the therapeutics and vaccines at our disposal. When the next influenza pandemic strain emerges, controlling its spread will be determined by our ability to treat infected patients and prevent others from becoming infected. Although no one-size-fits-all treatments are available, either therapeutically or prophylactically, the development of such treatments is being actively investigated.

Until recently, only three useful antiviral therapeutics have been licensed for use within the United States: oseltamivir, zanamivir, and peramivir. All three are NA inhibitors and are most effective when taken early in infections^71^. Though these therapeutics have been beneficial, an expanded repertoire of therapeutics and therapeutic classes is needed to effectively combat seasonal and pandemic influenza strains. For this reason, new treatment strategies are being explored and developed. For example, baloxavir targets the endonuclease activity of the influenza acidic polymerase protein, thereby preventing the polymerase from performing its essential cap-snatching function and disrupting influenza gene transcription^72^. However, mutations that confer resistance to baloxavir have already been detected, specifically, a mutation causing an I38T substitution in the acidic polymerase protein. An isolate with this mutation was reported in a patient treated with baloxavir in Japan^73^. Although baloxavir is still promising, the emergence of resistant influenza strains must continue to be monitored, and additional therapeutics must be developed.

The best defense against seasonal or pandemic influenza is vaccination. Currently, seasonal vaccines are administered annually; these vaccines contain H1N1, H3N2, and influenza B antigens, with a standardized amount of HA as the primary antigenic target^74^. These vaccines are specific to strains with minimal cross-reactivity and confer 40% to 60% protective efficacy when well matched^75^. However, limited cross-reactivity decreases the likelihood that seasonal vaccines can protect against pandemic strains. Furthermore, the vaccine production process is not ideal during a pandemic. Isolating a pandemic strain, growing it into a seed stock, propagating it into large quantities, inactivating and purifying it, and then mass distributing it is a lengthy and cumbersome process. Further, if the pandemic strain is highly pathogenic, biosafety regulations mandate their handling in high-containment facilities, limiting manufacturing capacity. It is possible to genetically modify these highly pathogenic viruses to where they can be handled at lower containment, but the associated process and required testing add complications and time to the vaccine production timeline. Two approaches may be used to engineer more effective vaccines: (1) universal influenza vaccines that induce broad cross-reactivity against many influenza subtypes and (2) vaccine platforms that are scalable and can be rapidly produced in response to an emerging novel strain.

Universal influenza vaccines are currently being investigated^76^. Multiple antigenic targets for a more broadly protective vaccine are being explored, all with varying benefits and challenges. One such target is NA. Anti-HA and anti-NA antibodies both correlate with protection from infection and disease, with anti-NA antibodies serving as an independent correlate of protection^77,78^.

Anti-HA antibodies are most strongly associated with preventing infections, whereas anti-NA antibody titers correlate with reduced amount and duration of viral shedding and reduced duration and severity of symptoms during infections^79^. Anti-HA antibodies typically have limited cross-reactivity because of their specificity to the HA head region, which undergoes regular antigenic drift in its epitopes^80^. Anti-NA antibodies confer a larger breadth of protection, with polyclonal sera generally having some limited inhibitory activity against NAs of the same subtype to which the sera were generated^81^. Although NA undergoes antigenic drift at a lower rate than HA, leading to more conserved epitopes across strains and broader protection^82,83^, whether this rate is maintained with widespread NA-based vaccinations and thereby focused immune pressure on NA is unclear.

The HA stalk is another antigenic target under investigation. Although the head of HA undergoes antigenic drift at a considerable rate and is highly variable across subtypes, the HA stalk region is more conserved. Broadly neutralizing anti-HA stalk antibodies have been isolated from humans, but these antibodies comprise only a minor fraction of polyclonal sera^84^. Anti-HA stalk antibodies may be generated by vaccinating with the HA stalk protein lacking the globular head because the head domain is immunodominant^85^. However, the removal of the head of the HA protein may result in improper conformation and glycosylation of the stalk and, therefore, the induction of antibodies that do not appropriately recognize wild-type epitopes^84,85^. Immunization with chimeric HA proteins composed of the stalk region of the H1 and H3 subtypes matched to novel avian head domains that humans are naïve to may circumvent this limitation, thereby selectively directing the antibody response to the HA stalk while priming anti-avian antibodies^84,86^. This strategy has promise, but age-dependent differences in anti-HA stalk immunity, HA stalk escape mutants, and potential autoreactivity of anti-HA stalk antibodies all warrant further exploration before this method of vaccination is widely implemented^87–89^.

Until a universal influenza vaccine is developed, a more practical approach to pandemic preparedness may entail developing scalable platforms to produce a pandemic vaccine quickly. One such platform is the production of mRNA-based vaccines. Conceptually, mRNA-based vaccines are quite simple; upon the administration of an mRNA encoding the vaccine target, the mRNA is internalized, and the host cell machinery then translates it into a protein that is both displayed on the cell surface for immune recognition and passed through the proteolytic pathway for peptide presentation. These vaccines are advantageous, particularly in the context of pandemic responses, because the production process is rapid and scalable, uses in vitro reactions (rather than embryonated chicken eggs), and can begin as soon as the antigen nucleotide sequence is known. Furthermore, the immunogenicity of mRNA-based vaccines may be adjusted by incorporating synthetic nucleotides and encapsulating them in various delivery vehicles to improve cellular uptake^90,91^.

Early studies of mRNA-based vaccines for influenza have shown promising protection profiles in mice, ferrets, and swine against a range of influenza strains^92^. The use of mRNA-based vaccines in pandemics is particularly highlighted by the SARS-CoV-2 pandemic. Multiple groups are developing mRNA-based vaccines for SARS-CoV-2. Moderna generated a vaccine with an mRNA encoding the SARS-CoV-2 spike protein mRNA-1273. Only 66 days occurred from the publication of the spike protein sequence to the enrollment of the first phase I clinical trial participant, a record-breaking timeline^93^. The mRNA-1273 vaccine is currently being tested in a phase III clinical trial, and preliminary results from the phase I trial show robust antibody responses to the vaccine in humans^94^. Outstanding questions of safety and efficacy remain, but those questions will most likely be answered in the coming months. Because mRNA can be used to produce any antigen, provided the sequence is available, this strategy may be particularly useful for influenza control. This strategy can be leveraged not only for pandemic responses but also for seasonal vaccine production, decreasing the time between strain selection and vaccine administration, thus providing more flexibility for strain selection.

### The role of industry in pandemic responses

Although preparation for the next influenza pandemic falls under the purview of government-led surveillance and research, the SARS-CoV-2 pandemic demonstrated the value of industry responses when pandemic-causing viruses emerge. Pharmaceutical companies contribute considerable resources to develop antivirals, including monoclonal antibody therapies from Regeneron and Eli Lilly currently under investigation in phase III clinical trials^95^. Additionally, Moderna, Johnson & Johnson, and AstraZeneca are investigating vaccines in phase III clinical trials^96^. All of these treatments were developed and tested preclinically and clinically in collaboration with university and government research institutions, illustrating the value of these collaborations. Maintaining such partnerships will better position us to respond to future pandemics.

### Public health messaging

A critical component of pandemic management is coherent and cohesive public messaging. The SARS-CoV-2 pandemic highlighted the inadequacy of scientific communication to the public, resulting in mixed messaging and unclear public health guidance. Science is an iterative process; therefore, guidance and conclusions about SARS-CoV-2 pathology and management evolved as information became known. However, scientific conclusions are presented as fact rather than ever-evolving ideas, which leads to public misunderstanding of how the scientific process is conducted and the perception that experts are changing their minds. A critical component of preparing for the next pandemic, influenza or otherwise, will be restoring faith in science. We must be transparent in how we perform science and communicate our findings. Furthermore, we must be united in presenting the conclusions that only sound evidence suggests. As we cope with the effects of the SARS-CoV-2 pandemic and prepare for the next pandemic, we must restore trust in our institutions or risk unmitigated loss of human life and faith in science.

### Intersection with SARS-CoV-2

Before SARS-CoV-2 emerged in late 2019, most of the influenza research community anticipated that the next pandemic would be caused by a novel influenza strain^97^. The SARS-CoV-2 pandemic raises questions about our overall pandemic preparedness and allows some reflection on the overlap of influenza risk assessments and other viruses with pandemic potential. The SARS-CoV-2 pandemic also serves as a model to evaluate our current pandemic workflow for its strengths and weaknesses.

A vital aspect of pandemic preparedness is surveillance. Influenza surveillance infrastructure and networks continuously monitor circulating strains in animal populations and identify prepandemic strains before they cross the species barrier. The GISRS has identified several prepandemic influenza strains, potentially providing a crucial advantage to prepare before the next influenza pandemic emerges and to contain such prepandemic strains.

Such networks were much less developed for SARS-CoV-2 because coronaviruses were identified only in the past two decades as potential pandemic-causing viruses^97,98^. The SARS and MERS outbreaks led to increased research on and surveillance of coronaviruses, but the time and resources to develop these surveillance and response frameworks have not been as extensive as those for influenza. This raises questions about the use of our resources, especially now that we fully understand the pandemic potential of noninfluenza viruses. Can we add to our current influenza risk assessment framework to account for other prepandemic viruses? The expertise and infrastructure developed over the past 70 years for influenza surveillance may serve as a model for coronavirus surveillance, if not directly incorporate it. We should also develop methods to identify other virus families with pandemic potential and consider expanding our surveillance to include these viruses.

Another aspect of pandemic preparedness is the ability to respond once a pandemic-causing virus emerges. For influenza, this response has two major categories: vaccines and antivirals. Routine production of seasonal influenza vaccines provides the knowledge and infrastructure needed to quickly develop, test, and produce pandemic vaccines. Prepandemic vaccines are also stockpiled for strains at high risk of causing pandemics^32,60^. Antiviral research has uncovered resistance mechanisms of different influenza strains and optimized therapies^99,100^. However, coronavirus-specific antivirals were not developed before the SARS-CoV-2 pandemic^101^.

Although the effort to develop a vaccine for SARS-CoV-2 is unprecedented and will most likely produce multiple vaccines in record time^49^, no coronavirus vaccine platforms were approved before the pandemic. Fortunately, previous and current work on SARS and MERS vaccines accelerated vaccine production, but novel noninfluenza pandemic-causing viruses will require more time for vaccine development. New vaccine platforms, such as mRNA vaccines^90,91^, which can be applied to an array of antigens, may decrease the time required for vaccine production in response to pandemics of almost any viral origin^48^.

Because influenza research has generated many laboratory tests to identify the presence of influenza and its subtypes, we are well prepared to quickly develop testing for novel strains that emerge, especially for prepandemic strains detected in influenza surveillance^54,102^. Human cases of prepandemic strains are quickly identified and closely monitored to contain any potential widespread outbreaks that may lead to a pandemic. However, when SARS-CoV-2 emerged, diagnostic testing kits required months to develop and widely distribute across the United States, which led to delayed case identification and contact tracing. Such delays are disastrous for containing or mitigating viral transmission. Therefore, the surveillance of all viruses with pandemic potential is crucial for pandemic preparedness. Testing should be developed for all potential classes of pandemic-causing viruses and be easily adapted to identify specific species or strains.

The three key lessons learned from the SARS-CoV-2 pandemic are (1) to continue and enhance the surveillance of prepandemic viruses, (2) to generate a method/platform for rapidly developing and producing pandemic vaccines, and (3) to implement strategies for prompt diagnostic testing responses. Our experience with SARS-CoV-2 will hone our pandemic preparedness workflows, including those for influenza, to better meet the challenges of pandemics in the future.

## Conclusion

Over the past hundred years, influenza has caused four global pandemics. The risks of influenza viruses are well known, and constant genetic drift and shifts in the influenza genome pose a continual threat to novel emergent strains. The 2009 H1N1 pandemic exposed weaknesses in our pandemic preparedness that have since been improved by increased surveillance, dedicated influenza research, and pandemic risk assessment tools. Determining which strains pose the most risk and laying out systematic workflows for resource allocation and research emphasis are now priorities.

Identifying and monitoring prepandemic influenza viruses such as H7N9 suggest that this workflow is functioning well and will give us an advantage should one of these viruses transition from prepandemic to pandemic. Continual routine influenza surveillance in animal populations known to be reservoirs for influenza and understanding the evolution of influenza viruses may reveal important patterns in mutations and recombination events to consider. However, the threat of a completely new strain emerging that is not currently on our radar is always present, highlighted by the SARS-CoV-2 pandemic. We must continually evaluate and update our pandemic workflows to stay ahead of the next deadly strain. With diligence, countless lives may be saved when the next pandemic arrives.
